# Supplementary material for: Correlations between the selection of topics by news media and scientific journals
Source: PLoS One. 2023 Jan 25;18(1):e0280016. doi: 10.1371/journal.pone.0280016 (PMC9876210; doi:10.1371/journal.pone.0280016)
Supplement: Attachment — Submitted filename: Response to Reviewers.docx [file pone.0280016.s001.docx]

**Response to Reviewers**

**Manuscript ID: PONE-D-22-17799**

Dear editors, dear anonymous reviewers!

We would like to thank all of you for the helpful feedback concerning the article we submitted to PLOS ONE. We have taken the editor’s and reviewers’ comments into account and hope that our changes and explanations are understandable and satisfactory. While revising our article, we felt that it gained in quality and we hope that you feel the same way when reading this letter and the revised article.

Below, we list the reviewers’ comments sorted by topic and address each point.

**Causality issue, accompanied by methodological problems**

Reviewer 1, comment 2: Reviewer 1 notes that our theoretical argumentation and hypotheses suggest causal inferences that we cannot prove with the analysis methods we used.

Reviewer 2, comment 2: Reviewer 2 also states that our analysis methods are too simple to test our assumptions. Comparative analyses/ control groups are required.

Reviewer 1, comment 5; Reviewer 2, comment 2: Both reviewers demand significance tests.

Reviewer 1, comment 1: Reviewer 1 further recommends the inclusion of social media data sources to be able to compare if they also have an influence on the topic selection of scientific journals.

Action taken:

We have taken these criticisms very seriously, because they are of a substantial nature for our paper. We agree with the reviewers’ critique. We decided that the agenda setting approach, which assumes causal effects of the media agenda on the scientific agenda, leads in the wrong direction. Therefore, we reframed our theoretical argumentation. We substantially revised chapters 1 (Introduction) and 5 (Summary and discussion), deleted chapter 2 (Feedback effects of media coverage on scientific publishing) and have reformulated hypothesis 1. We now explain that we consider journalistic coverage as an *indicator* of the social popularity of a topic and focus on the question whether socially popular topics (that have attracted public attention through media coverage) are considered worthier of publication by scientific journals than those which did not gain public attention. Primarily, we seek to plausibilize the existence of a robust connection between topic selection in journalism and research. In our new introduction, we try to make a plausible case that the selection criteria of quality journalism and scientific journals should be similar (earmark hypothesis), namely that both should be oriented toward socially relevant topics. And if this is the case, then one cannot separate a publicity effect from the earmark hypothesis without doubt, because according to the publicity effect, scientific journals focus on topics that are publicly relevant – which is also the reason why news media dedicate themselves to these topics. Ultimately, the distinction between the earmark hypothesis and the publicity effect is of secondary importance in our view. What matters is that there is a relationship between the choice of topics in journalism and in scientific journals.

To make the new theoretical focus of our paper clearer, we have also changed the title of our manuscript to “Correlations between the topic selection of news media and scientific journals”.

Based on our methodological approach, as the reviewers correctly note, causal interpretations cannot be deduced without doubt. Hence, we focus our analysis and interpretation to *correlations* between the selectivity of journalism and that of research journals. We enriched our analyses with significance tests. Because of the skewed distribution of ratios with strong outliers, we opted for a nonparametric approach and thus fortwo-sample Wilcoxon tests. The analysis of Figure 3 serves as a control group design and is now explicitly named as such.

The inclusion of social media data sources to test whether they have an influence on the topic selection of scientific journals is no longer necessary due to the new theoretical frame. However, in order to take up this point in substance, we discussed in the manuscript (chapter 1) the strong mutual coupling of agendas in journalism and social media, which makes it extremely challenging if not impossible to isolate a journalistically mediated public sphere and its impact from that of social media. This serves as a further argument why we refrain from a causal view.

**Effects of media coverage on citation rates**

Reviewer 1, comment 3: Reviewer 1 does not entirely agree with our argument about effects of media coverage on citation rates.

Explanations:

Due to our new theoretical framework, the objection in comment 3 by reviewer 1 becomes obsolete, since we no longer make the criticized argument. We do not talk about effects of media coverage (on citation rates) anymore.

**Precision degree of the “similar articles” function of PubMed**

Reviewer 1, comment 4: Reviewer 1 wonders, how much of an impact the rather weak precision of the “similar articles” function of PubMed has on our results.

Explanations: We consider the quotient of the number of similar papers before and after a point in time. A low precision goes into the numerator as well as into the denominator, so the result remains expectation-true, albeit with a higher variance. As long as the precision of the “similar articles” function remains stable over time, it has no influence on the result of our analysis. There is no plausible reason to assume that precision should change over time.

**Threshold of MSM-score**

Reviewer 2, comment 3: Reviewer 2 expects a more detailed justification of why we chose the threshold more than or equal to 100 for MSM-scores. Furthermore he/she suggests to select and test different thresholds.

Explanations:

In a previous study, we validated the MSM-score score to determine the level at which the MSM-score could be interpreted as an indicator of noteworthy popular media coverage of a scientific research article. Our validation was based on 1,601 scientific articles that were published in the journals Nature and Science between January and October 2017 (extracted from Scopus database). We collected the MSM-scores of these studies and assigned them to 11 groups, namely scores of 1–9; 10–19; 20–29;…; ≥ 100. Subsequently, we conducted a manual search of randomly selected sets of five research articles per group (N = 55) in the full-text press database Nexis to determine from which score we could infer journalistic coverage on three large national media markets (the United Kingdom, the United States, and Germany). The amount of press coverage was classified as ‘noteworthy’ if at least 19 articles on at least one of the five studies per category had appeared in these media markets. A previous study validated this score and Our results showed that only a score ≥ 50 indicates that single media titles pick up a scientific paper. From a score of ≥ 100 can it be assumed that a result has been taken up congruently by a larger number of media titles in different countries (USA, UK, and Germany), indicating a broad international dissemination [28]. We rely on this value ≥ 100 as a cutoff value to determine our population […].

Action taken:

We have integrated this text passage into our manuscript (chapter 4.1).

In addition, we have pointed out the following limitation of our study in chapter 5: “Another uncertainty concerns the threshold of the MSM-score, which we defined as an indicator of noteworthy popular media coverage of a scientific research article. We defined this threshold on the basis of a validation study conducted by us. However, we cannot say with absolute certainty that the MSM ≥ 100 papers do not include papers that have not received noteworthy international media coverage. Ultimately, we cannot provide a very specific measure of when a scientific study has actually received broad international media attention.”

Additional explanations:

We do not use the MSM-scores to conduct any statistical analyses, but only as a cutoff variable that indicates, which research article received popular media attention. We treat the MSM-score as a categorical variable: Either a study has received relevant media attention, or it has not. Therefore, we see no need to test several values.

**Accumulation of the MSM-score**

Reviewer 2, comment 1: Reviewer 2 thinks that the time it takes for the MSM-score to develop is problematic for our data collection and processing method.

Explanations:

Reviewer 2 considers that „[i]t takes some time for the MSM-score to accumulate to a high number.” Therefore, our data collection and processing method seems problematic for him/her.

We would like to make two counterarguments:

1) We do not share the assumption that it takes about (more than) a year until an MSM-score reaches 100 points. We found several literature references which state that “Altmetric scores react immediately after publication of a study.” [1] See also [2]: “[research] articles may be mentioned predominately during the time period that they are first published.” Also, reviewer 1 argues, that MSM-scores “are accumulated in the early stage after the publication of papers.” The reviewers contradict each other on this point.

Also, from a communication science perspective, it makes entirely sense that news media pick up a scientific study when it is freshly published. When it comes to the question of what is newsworthy, news media and journalists are guided by so-called news factors, among other things (see news value studies, e.g. [3]). One crucial news factor with a strong impact on the selection process of science news is “actuality” [4]. Only rarely are studies that were published months or years earlier selected for science news coverage – for example, if they can be thematically re-updated.

Literature:

1. Studenic P, Ospelt C. Do you tweet? Trailing the connection between Altmetric and research impact! RMD Open. 2020;6(3): e001034. doi: 10.1136/rmdopen-2019-001034

2. Elmore, SA. The Altmetric attention score: What does it mean and why should I care? Toxicologic Pathology. 2018;46(3): 252-255. doi: 10.1177/0192623318758294

3. Maier, M, Stengel, K, Marschall, J. Nachrichtenwerttheorie [News value theory]. Konzepte. Ansätze der Medien- und Kommunikationswissenschaft. Baden-Baden: Nomos; 2010. doi: 10.5771/9783845260365

4. Badenschier F, Wormer H. Issue selection in science journalism. Towards a special theory of news values for science news. In: Rödder S, Franzen M, Weingart P, editors. The Sciences’ Media Connection: Public Communication and its Repercussions. Dordrecht: Springer; 2012. pp. 59–85.

2) Irrespective of whether or not one may follow our argumentation under 1), from our point of view there is no problem with the data collection for the following reason: We retrieved the MSM ≥100 paper all at a consistent point of time, namely in May 2019. Since the publication date of our selected papers had to be between August 2014 and July 2018 (pick-up criterion), the collection date is far enough behind the publication date of the articles, so that an MSM-score should already have developed. The MSM-score ≥ 100 is a cutoff value for us to determine the population: We have only collected papers that already have generated an MSM-score ≥ 100. We are not further interested in the development of the MSM-scores of the selected papers and do not make any analyses with the MSM-score. It solely serves as a criterion for the selection of our population.

Action taken:

We now clarify the use of the MSM-score as a pure cutoff value to determine the population in our manuscript by using the following wording: “We rely on this value ≥ 100 as a cutoff value to determine our population and thus identified all scientific papers with an MSM-Score ≥ 100 that were published during a time period from August 2014 to July 2018 by applying an automatic search on *Altmetric* using a validated Python script [37]. The collection of all scientific papers that met the cutoff criterion took place in May 2019. These were 1,068 scientific articles from 261 scientific journals.”

**Dataset figure 3**

Reviewer 1, comment 6: Reviewer 1 wonders why our dataset related to figure 3 consists of only two instead of six opinion-leader journals.

Explanation:

The analysis which relates to figure 3 serves as a control group design to test for effects of highly prestigious scientific journals: We ask if scientific papers that were originally published in highly renowned scientific journals but have *not* gained popular media attention imply an increase of similar articles in other scientific journals? The idea behind the limitation of our analysis to the journals *Nature* and *Science* is the following: If we were able to find a journal reputation effect, it would be in these two journals, since they represent the most prestigious scientific journals. Adding other but less prestigious journals would rather water down the results.

Action taken:

We have included this explanation into our manuscript: “The idea behind the limitation of our analysis to *Nature* and *Science* is the following: If we were able to find a journal effect, it would be in these two journals, since they represent the most prestigious scientific journals. Adding other but less prestigious journals would rather water down the results.”

**Typos**

Reviewer 1, comment 7: Reviewer 1 complains about typos.

Action taken:

We have thoroughly proofread the manuscript and hope to have eliminated all typos.

**Additional requirements, correct implementation of PLOS ONE’s style guidelines**

Action taken:

We have implemented all noted journal requirements and have reviewed the manuscript with regard to the application of the journal's guidelines.
