# Supplementary material for: Correlations between the selection of topics by news media and scientific journals
Source: PLoS One. 2023 Jan 25;18(1):e0280016. doi: 10.1371/journal.pone.0280016 (PMC9876210; doi:10.1371/journal.pone.0280016)
Supplement: Attachment — Submitted filename: Response to Reviewers.docx [file pone.0280016.s002.docx]

**Response to Reviewers**

**Manuscript ID: PONE-D-22-17799**

Dear editor, dear anonymous reviewer!

We would like to thank you for the positive feedback on our article “Correlations between the selection of topics by news media and scientific journals”. We have taken the editor’s and reviewer’s final comments into account and hope that our changes are satisfactory so that nothing stands in the way of a publication of our mauscript.

Below, we list the editor’s and reviewer’s comments and address each point.

**Abtract**

The editor suggests to include the following aspects into the abstract: details related to the sample (including the n), population and methods of study, key statistics to support the key findings (if they can stand alone), and a conclusion that sums up the key relevance.

Action taken:

We have included all these apsects in our abstract.

**Introduction**

The editor suggests to rework the introduction (remove repetitions, succinctness, reformulation of individual sentences).

Action taken:

We have reworked, partly reformulated and shortend our introduction and tried to highlight the important aspects more terse. Additionally, we had a proofreader correct the manuscript so that linguistic and grammatical errors and/or ambiguities should be eliminated.

**Copy editor**

The edditor recommends having a copy editor work through the manuscript for conciseness and clarity in sentence structure.

Action taken:

To improve the writing and to meet grammatical issues, we had the paper proofread by a professional proofreader (native speaker), who is not an expert on the topic of our paper. We further explicitly asked her to draw our attention to passages where connections are not clear or where further information is needed so that we can fix any problems.

**Potential time bias**

Reviewer 2 expects time bias in our data related to the time of publication of our analyzed scientific papers.

Explanations:

We thought about what kind of time bias the reviewer expects.

Version 1:

The number of MSM ≥100 articles in our sample is not evenly distributed over the analyzed publication years. In 2014 and 2015, for example, we have significantly fewer MSM ≥100 articles in the sample than in the years 2016-2018. The distribution looks like this:

| **Publication year** | **Number of MSM ≥100 articles**  (for which we could derive similar articles via PubMed) |
| --- | --- |
| 2014 | 15 |
| 2015 | 62 |
| 2016 | 373 |
| 2017 | 340 |
| 2018 | 193 |

Thus, there is time bias in our data. We suspect that this is because, over the years, Altmetric has changed respectively expanded the source base they track to capture references to scientific papers. Of course, we cannot change these different numbers of hits or cases. However, we do not consider this to be a problem for our analyses for the following reasons:

1) We have not carried out any stratified analyzes broken down by year of publication, otherwise our database per year would also be too small. However, we carried out time-sensitive analyzes insofar as we analyzed the development of similar articles depending on the year of publication of the MSM ≥100 articles (the two years before and the two years after the year of publication). Thus, we have already calculated the number of similar articles depending on the publication year of the MSM ≥100 articles.

2) We have checked whether the different case numbers in the individual years have an impact on our results. An analysis using only the MSM ≥100 articles from the years 2016-2018 (excluding the years with the low number of cases) resulted in no change in our key findings. We are still seeing the same trends and the findings that were previously significant continue to be significant. Our findings thus remain relatively stable, which is plausible as only relatively few cases are excluded from the analysis.

Version 2:

The reviewer may fear a possible bias due to an assumed dynamic development of the MSM-scores even more than two years after publication. Already in our first revision, we argued against such a dynamic development of MSM-scores for a longer period of time (more than two years) and referred to two relevant publications. We cannot retrospectively reconstruct the development of the MSM-scores of the articles we analyzed. But even if the MSM-scores were to continue to develop more than two years after initial publication, we wouldn't see any problem for our data analysis. This is because we do not carry out any comparative analyzes in which the MSM-score represents an independent variable. We use the MSM-score as a pure, binary cutoff criterion that is intended to state whether a scientific paper has received noteworthy media attention or not. So it makes no difference for our analysis whether a scientific article has an MSM-score of 100, 150, 200 or 250 etc. If we should miss some MSM ≥100 articles in recent years of publication (e.g. 2018) because they have not yet developed an MSM-score ≥100 at the time of data retrieval (which is unlikely), this is not a problem as long as they are missing at random. A systematic absence does not seem plausible to us. And even if they were systematic, in order to change our central findings, we would have to expect that the correlations we have detected for those MSM ≥100 articles, which only develop an MSM-score late (after more than two years), behave differently or exactly the opposite way compared to those MSM ≥100 articles that have achieved an MSM-score of 100 or higher within two years after publication. This does not seem plausible.

Action taken:

We have mentioned and discussed the uneven distribution of MSM ≥100 articles over the publication years in our limitations.

We have further tried to make even more explicitly clear, that we did not carry out any comparative analyzes in which the MSM-score represents an independent variable.

**Additional changes**

Action taken: According to the proofreader’s suggestion we have changed the axis label of Figure 2.
